# Safe environments—Through the eyes of 9‐year‐old schoolchildren from a socially vulnerable area in Sweden

**DOI:** 10.1111/cch.12809

**Published:** 2020-09-29

**Authors:** Karin Enskär, Gabriella E. Isma, Margaretha Rämgård

**Affiliations:** ^1^ Department of Care Science, Faculty of Health and Society Malmö University Malmö Sweden; ^2^ The Health Promotion Platform in Collaboration Malmö University Malmö Sweden

**Keywords:** participatory design, safe environment, schoolchildren, socially vulnerable area

## Abstract

**Background:**

Children are more vulnerable than adults to environmental risks. Also, children have little control over their environment. Unlike adults, they may be both unaware of risks and unable to make choices to protect their health. Children living in especially vulnerable areas might be even more at risk due to socio‐economic factors, immigration and high crime rates. Therefore, the aim of this study was to describe the perceptions that schoolchildren from a socially vulnerable area have of safe environments.

**Methods:**

Fifty‐two 9‐year‐old schoolchildren from a socially vulnerable area participated in this study. The data collection consisted of an environmental walk with photovoicing, followed by rating of the photos and a focus‐group discussion elaborating on the photos and ratings. Six focus groups, with six to eight children in each group, were conducted and analysed using inductive content analysis.

**Results:**

The results show that, according to the children, places that they think are bright and beautiful, where they can do fun things with others and do not risk being exposed to danger, create safety. To increase safety, the children suggested cleaning up, making the environment beautiful with grass and flowers and painting it in nice colours. Furthermore, they suggested that building features that increase the opportunities to play and engage in activities together with others would improve safety and enhance protection and surveillance.

**Conclusions:**

All children have the right to protection and safety. Therefore, it is important to create safe environments for all children by listening to children's own voices.

## 1 INTRODUCTION

Children are more vulnerable than adults to environmental risks, both physical and psychosocial, because of factors related to the development and growing up. Moreover, children have little control over their physical and social environment. Unlike adults, children may be both unaware of risks and unable to make choices to protect their health. Comprehensive risk assessments suggest a cluster of environmental hazards, many of which may concur in the places where children dwell, play and learn (WHO, 2020). In addition to studies that examined the role of environmental qualities in child development, more work is needed on the developmental impacts of the physical environment (Evans, 2006). Also, little is known about the role of cumulative exposure to multiple environmental conditions upon children. Children from low‐income areas are exposed to multiple suboptimal physical and social environmental conditions (Evans, 2004) that portend adverse developmental impacts (Repetti, Taylor, & Seeman, 2002; Taylor, Repetti, & Seeman, 1997).

It is necessary to look upon the future on children's safety from a new angle. Giving children and young people a greater voice in decisions affecting their everyday lives and considering their social background are important factors in improving their safety (Långberg, 2005). There is a lack of knowledge about schoolchildren's own perception of safe environments, and it is, furthermore, of utmost importance that good and successful child safety work covers all children in Sweden, regardless of socio‐economic circumstances.

## 2 BACKGROUND

In 1989, the General Assembly of the United Nations (UN, 1989) adopted and opened the resolution about the UN Convention on the Rights of the Child (UNCRC) for signature, ratification and accession (UN, 1989). The implication of the convention is that all rights apply to all children without exception. Sweden was one of the first states in the world to ratify the convention in 1990. However, like other countries, Sweden has been criticized by Humanium (2020), an international child sponsorship nongovernmental organization (NGO), for implementing children's rights inconsistently. This means that some young people are less protected than others because they live in places where their needs are not sufficiently taken into account. Since 2000, immigration has increased considerably in Sweden, witch provoked a rise in racism and discrimination, to the detriment of children who belong to migrant families (Gerell, Hallin, Nilvall, & Westerdahl, 2020).

*Vulnerable populations* can be defined as individuals who face significant barriers to better health (AJMC, 2006) and who are significantly affected by housing, employment status, educational opportunities and other social determinants of health. The social determinants of health include the living conditions at the individual as well as on community and societal/environmental levels (Dahlgren & Whitehead, 2007) and include socioeconomics and culture as well as the natural environment. Migrants and ethnic minorities are seen as potentially vulnerable. In its broadest sense, social vulnerability is one dimension of vulnerability to multiple stressors, including abuse, social exclusion and natural hazards (Jeffrey, Siegel, Jorgensen, & Alwang, 2001). In 2017, the Swedish Police Authority released a report on so‐called vulnerable areas, including 23 areas considered especially vulnerable. An ‘especially vulnerable area’ is characterized by social issues and a criminal presence that has led to a widespread disinclination to participate in the judicial process and difficulties for the police to fulfil their mission (Brå, 2018, p. 9; Gerell et al., 2020; Polisen, 2017). Also, those areas have an infrastructure in decline and lack of investment in public amenities such as playgrounds (Gerell et al., 2020).

Every child and young person has the right to a *safe environment*. The UNCRC states that the child must have the opportunity of growing up and developing (Article 6) in an environment that is as healthy and safe as possible and that the child's guardians should be provided with information on how to protect the child (Article 5) (UN, 1989). WHO (2005) invites member states to appoint national focal points and to engage in the development of national action plans for reducing the burden of unintentional or intentional injury in children and young people. One of the priorities outlined by WHO (1986) in the Ottawa Charter for health promotion was to create physically and psychosocially supportive environments for children and adolescents. This chart has led Swedish health policymakers to set the explicit goal of integrating public health with general welfare policy (Kickbusch, 2003).

The ecological systems theory, by Bronfenbrenner (1979), states that human development is influenced by the different types of *environmental* systems. Children typically find themselves enmeshed in various ecosystems, from the most intimate home ecological system to the larger school system and then to the most expansive system, which includes society and culture. Each of these ecological systems inevitably interacts with and influences each other in all aspects of the children's lives (Bronfenbrenner, 1979, 2005). The ecological context has mainly focused on the psychosocial aspects of children's environments, largely ignoring the physical environment and its effect on child development, as the physical environment can influence child development directly and via adult caregivers (Evans, 2006). Even though many of the underlying processes that connect context to development are similar for physical and psychosocial environmental factors, as parent–child interaction and other interpersonal processes, self‐regulation, physiological adaptations and control beliefs are altered by the physical environment (Evans, 2006). A growing body of research documents significant effects of the physical environment (toxins, pollutants, noise, crowding, chaos, housing, school and neighbourhood quality) on children and adolescents' cognitive and socioemotional development (Evans, 2006; Ferguson, Cassells, MacAllister, & Evans, 2013). Also, the neighbourhood physical environment has a significant association with early childhood development (Bell et al., 2020).

*Safety for children* can be defined as the absence of risk or a low probability of being exposed to risks in their physical and social environment, meaning that the child is protected against danger and unwanted events and does not risk being physically and psychosocial harmed or killed. So far, the work for safe environments has mainly focused on the physical environment, decreasing injuries in children. Sweden has a long tradition of preventive child safety, which has led to one of the world's lowest numbers of children harmed and killed (Sethi, Towner, Vincenten, Segui‐Gomez, & Racioppi, 2008). The chances of children living in a safe physical and social environment are affected by a number of factors that also increase proneness to physical and psychosocial harm. Studies have linked higher risks for childhood unintentional harm to, for example, poverty, single‐parenthood, mothers' low level of education and low childbearing age, poor housing conditions, large families and parents' alcohol and drug use (Thomas et al., 2007).

Children who are exposed to optimal physical and social environments early in life have the best opportunities to grow up healthy and happy (Irwin, Johnson, Henderson, Dahinten, & Hertzman, 2007). On the other hand, adverse experiences early in a child's life can lead to poor health, poor educational attainment, economic dependency, increased violence and crime, substance abuse and depression (Chan, 2013). All of these add to the burden and cost to society, including the health system (Chan, 2013). Research shows that limiting exposure to physical and psychosocial harm and promoting protective factors in the early years can reduce the need for more costly interventions later in life (Karoly et al., 1998). Positive interventions in early childhood can also help mitigate the impact of adverse experiences. Also, the interplay between the neighbourhood environment and proximal influences, including socio‐economic status and how it influences early child development, need to be considered (Bell et al., 2020). Therefore, the aim of this study was to describe the perceptions of safe outdoor environments of 9‐year‐old schoolchildren from a socially vulnerable area.

## 3 METHOD

An explorative qualitative design with a participatory and inductive approach was applied because there is limited knowledge about children's experiences of safe environments. Photography and focus group interviews were used because these approaches are considered useful methods for exploring the children's own experiences (Gibson, 2007). Research with children from a participatory perspective has been advocated and used when children are participants in the projects (Davis et al., 2018; Einberg, Nygren, Svedberg, & Enskär, 2016; Gibbs et al., 2018; Larsson, Staland‐Nyman, Svedberg, Nygren, & Carlsson, 2018). This means that the participants play an active role throughout the project. In this study, the schoolchildren were an active part of the research process and not only participants in data collection.

### 3.1 Setting

The study is a part of a larger initiative for developing and studying health‐promoting activities informed by community‐based participatory research in the socially disadvantaged district ‘Lindängen’ in the city of Malmö. The urban area has 7,600 inhabitants, about 75% of whom are first‐ and second‐generation immigrants, many with a Middle Eastern origin. The area has been considered one of the most socially deprived areas in Sweden and meets the criteria for being an ‘especially vulnerable area’ (Brå, 2018, p. 9; Gerell et al., 2020; SCB, 2017; Swedish Police Authority, 2017). There is a high rate of unemployment and crime, low education levels, poor housing and poor health among the residents (Brå, 2018, p. 9; Gerell et al., 2020; SCB, 2017). Furthermore, child poverty is high, especially in immigrant families (Salonen, 2018; Save the Children, 2019). In the area, researchers from Malmö University, together with different organizations and local ‘health promoters’, run six health‐promotion activities created for integration through social meeting places in the residential area.

### 3.2 Participants

The study was carried out in a primary school in Lindängen, Malmö, Sweden. All children (*n* = 52) in their second school year (in 2018–2019) participated in the study, thus consisting of children aged 9 years, of whom 25 were girls and 27 were boys. The children had several ethnic and cultural backgrounds, being first‐ or second‐generation immigrants; none of the children had two parents born in Sweden.

### 3.3 Data collection and procedure

The data collection was based on a *participatory perspective*, and all children were active participants in the design and data collection. The teacher, in collaboration with a health promoter, led the data collection together with the children as part of their regular teaching at the school. An environmental walk with photovoicing was chosen, by the children, as data collection, with a following rating of the photos and a focus‐group discussion elaborating on the photos and ratings. Photovoice is a participatory method, defined as a process by which people can identify, represent and enhance their community through a specific photographic technique (Wang & Burris, 1997), previously used in research with children (e.g., Einberg et al., 2016). The children in this study were engaged participants in taking photographs symbolizing safety (Riley & Manias, 2004). Photographs can stimulate reflection and provide a basis for not only a discussion of what they illustrate but also a discussion of the underlying meanings (Coad, 2007). The children, in six groups (six to eight children per group), together with the teacher, the health promoter and representatives from Save the Children, conducted photovoice sessions during their walk in the area of Lindängen, taking photos with an iPad of places the children considered safe or unsafe, resulting in 48 photos from the six groups.

These photos were placed in a database at the school, and when all the duplicates were removed, 28 photos remained. The teacher and the health promoter showed all 28 photos, one by one, and each child rated the photos digitally with the help of emoji symbols, related to three categories: safe places 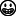, unsafe places ☹ and places that did not arouse any special feelings 😐. The use of emoji symbols as a visual research method is based on a type of graphic symbol that can be used to represent a facial expression but has been more widely co‐opted to represent feelings, gestures, objects and activities. This research method for engaging children in showing how they understand and make meaning of their world may offer both a practical and an insightful approach to eliciting young children's voices in childhood research (Fane, MacDougall, Jovanovic, Redmond, & Gibbs, 2018).

Thereafter, the children were divided into six groups (six to eight children per group) and *interviewed according to a focus‐group process* (Gibson, 2007). The groups were led by the health promoter, teacher and/or researcher. The children were shown one picture at a time, together with the ratings by means of the emoji symbols, and were asked: ‘Do you think this is a safe place or an unsafe place and why?’. To deepen the data, children were asked to elaborate on and clarify their statements. The interaction between the children provided in‐depth answers to the questions, which were illuminated from a variety of perspectives.

### 3.4 Data analysis

The analysis of the focus group interviews was based on the method of qualitative content analysis (Elo & Kyngas, 2008). The units of analysis were the transcribed interview data from the focus groups. To obtain a sense of the whole, the transcribed text together with the photographs (*n* = 14) were reviewed and read several times. The next step in the analysis was open coding, when notes were made in the transcribed material. A coding scheme with meaning units was established, and the initial work of creating codes and categories began. The authors had, on several occasions, a dialogue with each other regarding codes and categories to move the analysis forward and ensure trustworthiness. The analysis went back and forth between codes, subcategories and categories.

### 3.5 Ethical consideration

Ethical research with young children is facilitated by a multistep procedure for ensuring that the research design is suitable and reasonable and prioritizes the safety and security of the child participants. The research was based on the Helsinki Declaration 1964 (World Medical Association, 2013) and, prior to initiation, approved by the Ethical Review Board in Lund (Reg. No. 2018‐591). The school was part of the larger initiative for developing and studying health‐promoting activities in the area, including teachers, health‐promoters and the principal. All children and parents were verbally informed about the study, and parents gave their consent for their children's participation. A teacher from the school was present at the data collection and asked the children for assent by clearly explaining what the research activity would entail and took into account both verbal and nonverbal cues to ensure that the children had the opportunity to give their assent.

## 4 RESULTS

**FIGURE 1:**
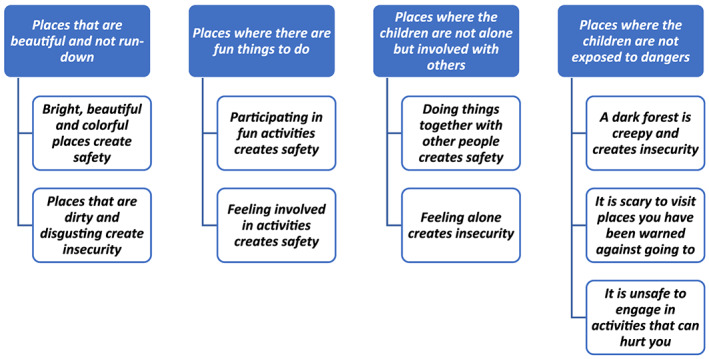
Children's views of safety in their environment [Colour figure can be viewed at wileyonlinelibrary.com]

During the focus‐group interviews, the children described safe places as (1) places that they think are bright and beautiful, (2) places where they can do fun things, (3) together with family and friends, and where they (4) do not risk being exposed to dangers; according to the children, such places create safety. In the analysis, four categories and nine subcategories describe the children's views on safe environments (Figure 1).

### 4.1 Places that are beautiful and not run‐down

**FIGURE 2:**
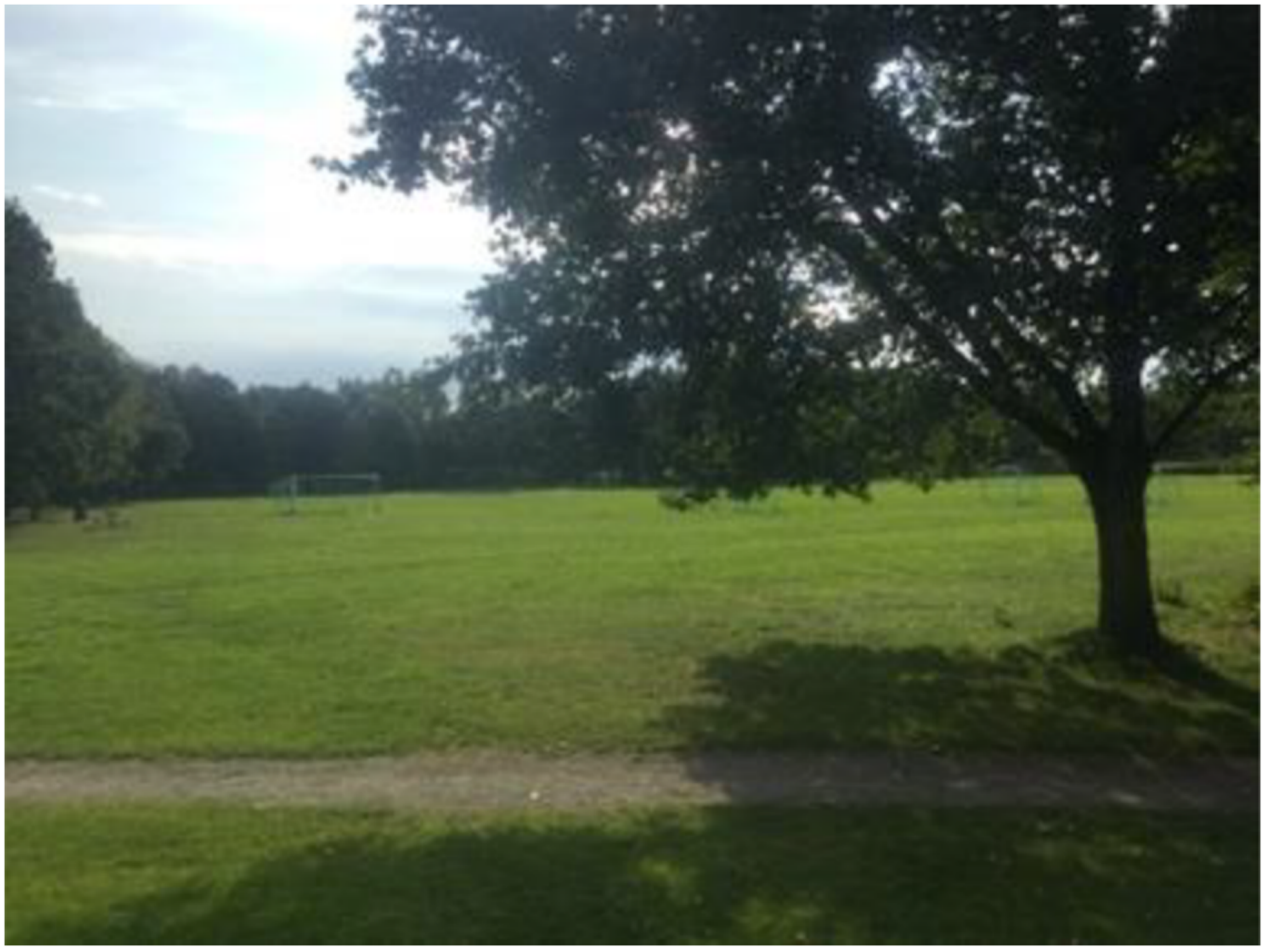
A bright and beautiful place [Colour figure can be viewed at wileyonlinelibrary.com]

**FIGURE 3:**
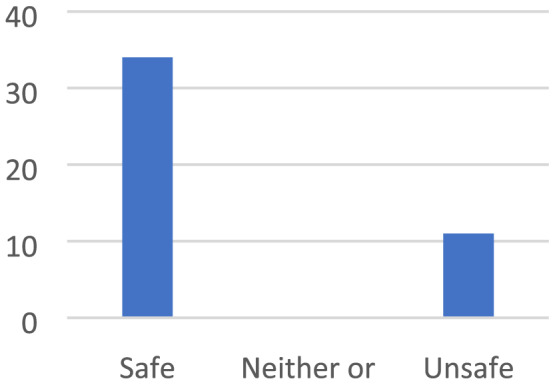
Ratings related to Figure 2 of a bright and beautiful place [Colour figure can be viewed at wileyonlinelibrary.com]

The children described feeling safe in places that are bright, beautiful and colourful. In the same way, the children described how being in places that are messy, dirty, disgusting and smelly felt unsafe (Figures 2 and 3).

#### 4.1.1 Bright, beautiful and colourful places create safety

**FIGURE 4:**
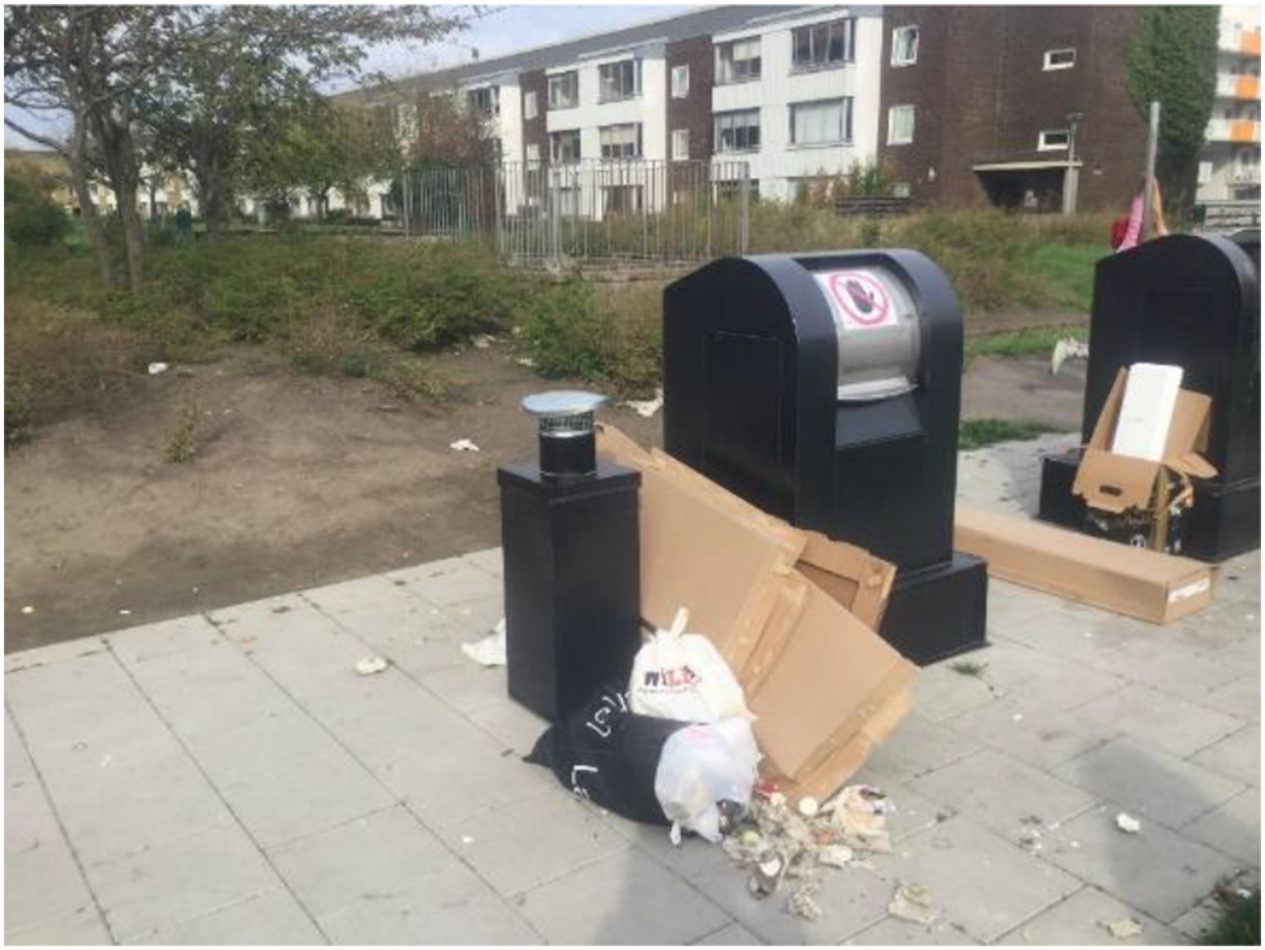
A dirty and disgusting place [Colour figure can be viewed at wileyonlinelibrary.com]

**FIGURE 5:**
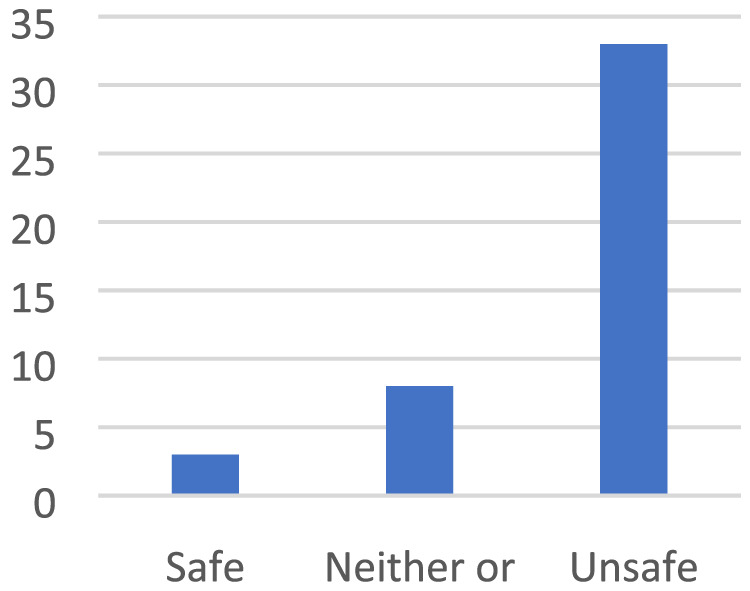
Ratings related to Figure 4 of a dirty and disgusting place [Colour figure can be viewed at wileyonlinelibrary.com]

The children described being in places that are bright, beautiful and colourful as safe. They enjoyed it when the sun sets without being obscured by bushes and trees. They perceived themselves as safer in the summer when it is bright and sunny outside: ‘A green place in summer when flowers grow’. Similarly, they said that places that have been deliberately made beautiful by planting grass and flowers feel cosy and safe: ‘It is bright and nice, lots of trees to climb, cozy, tread on nice grass’. A prerequisite for a place to be perceived as beautiful and safe was that it must be clean and not run‐down. To enhance and make the places cosier and safer, the children suggested making them beautiful with grass designs and flowers and painting walls and houses in nice colours, in fresh white and not brown, saying that ‘You can add flowers and add rainbow colors’ (Figures 4 and 5)

#### 4.1.2 Places that are dirty and disgusting create insecurity

In contrast to the beautiful places, the children described places where it was messy and smelled bad and where there was garbage as ‘scary place’. These places were described as unsafe by the children: ‘Unsafe, smells disgusting’. Regarding the garbage station, the children thought it was the adults' responsibility to throw garbage in the trash cans and to put on the lid. The children said that there was a lot of garbage attracting animals such as rats. They pointed out that it is important to prevent places from getting dirty by not throwing garbage: ‘People should not throw garbage, you can make signs so people know they should not throw garbage’. Moreover, the children said that it is the adults' responsibility to make sure the garbage station does not become dirty. But they also wanted to set up rules for waste separation and have signs telling people not to throw trash and for more trash cans to be arranged. In addition, the children suggested cleaning up, removing the ugly stuff, washing it away and cleaning up: ‘Washing and making things look nice’.

### 4.2 Places where there are fun things to do

**FIGURE 6:**
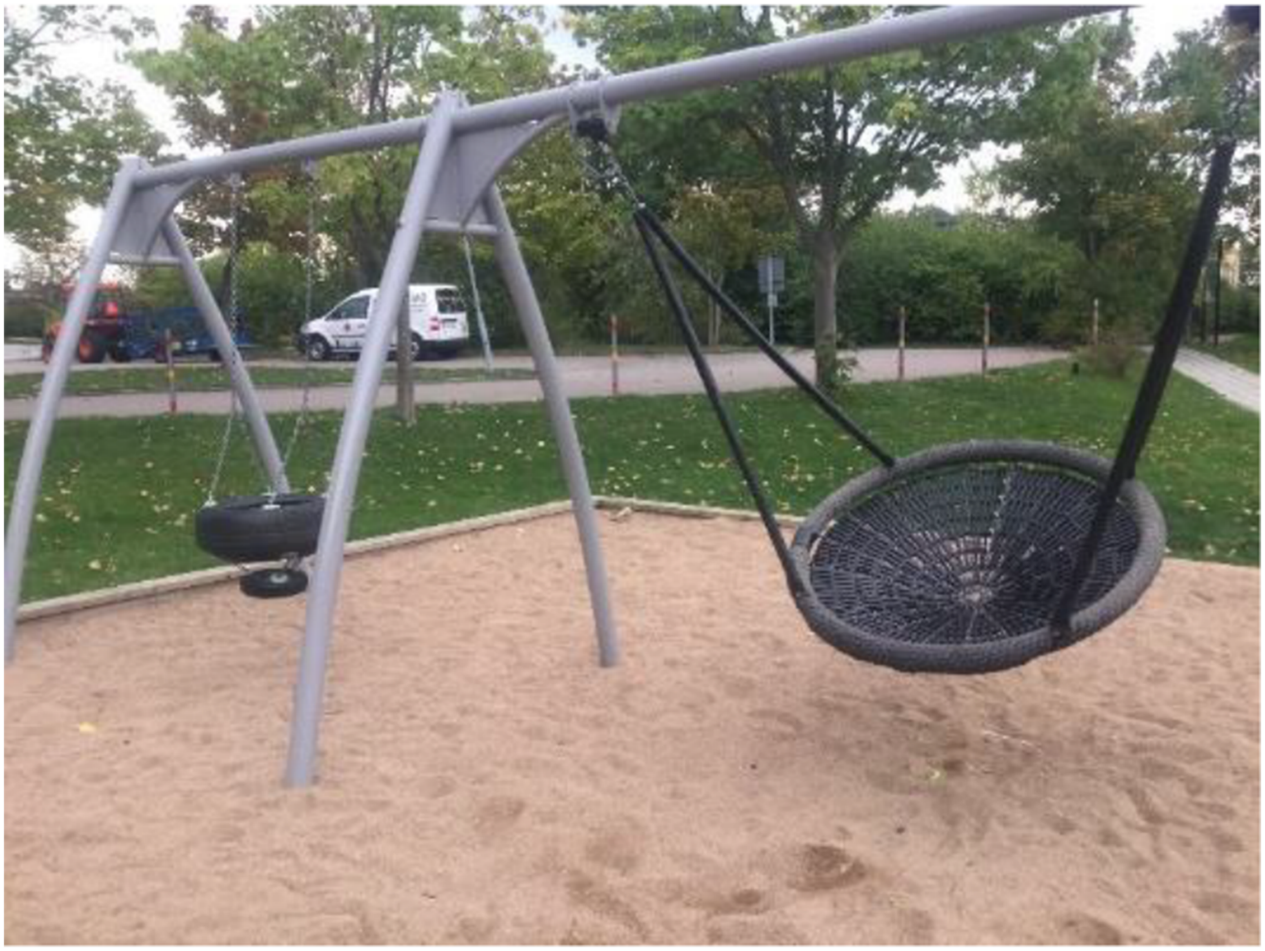
A place with fun activities [Colour figure can be viewed at wileyonlinelibrary.com]

**FIGURE 7:**
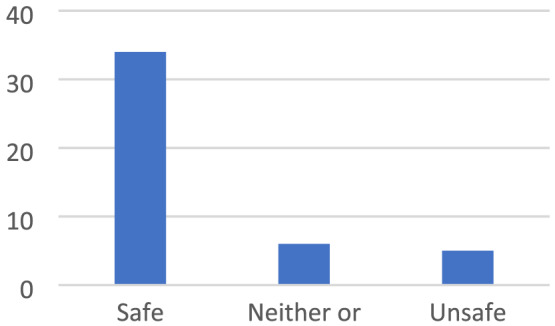
Ratings related to Figure 6 of a place with fun activities [Colour figure can be viewed at wileyonlinelibrary.com]

Having fun and participating in activities were described by the children as a way to feel safe. Such places for having fun could be the football pitch, the swings or the library (Figures 6 and 7).

#### 4.2.1 Participating in fun activities creates safety

The children talked about fun outdoor play, ‘You can play football, basketball’, such as playing football, swinging, playing in a playground or being in the outdoor gym, ‘Fun swing’. The children also talked about indoor activities such as theatre, reading books or playing games on the computer. Similarly, they described how they felt a lack of safety in places where there is nothing to do. To enhance safety, the children suggested creating opportunities for activities in the environment, such as a new playground with swings, ‘Build a playground around the center’, and slides, ‘Waterslide’.

#### 4.2.2 Feeling involved in activities creates safety

The children also described how they felt when more involved in activities, ‘You can borrow books in your own language’, and when they understood and could learn something from the activities, ‘You play football and you learn’; this created security. To create a safer environment for children to feel involved in, they suggested rebuilding and new arenas, such as a new and safer downtown, ‘Maybe to make it a secret underground downtown’.

### 4.3 Places where the children are not alone but involved with others

**FIGURE 8:**
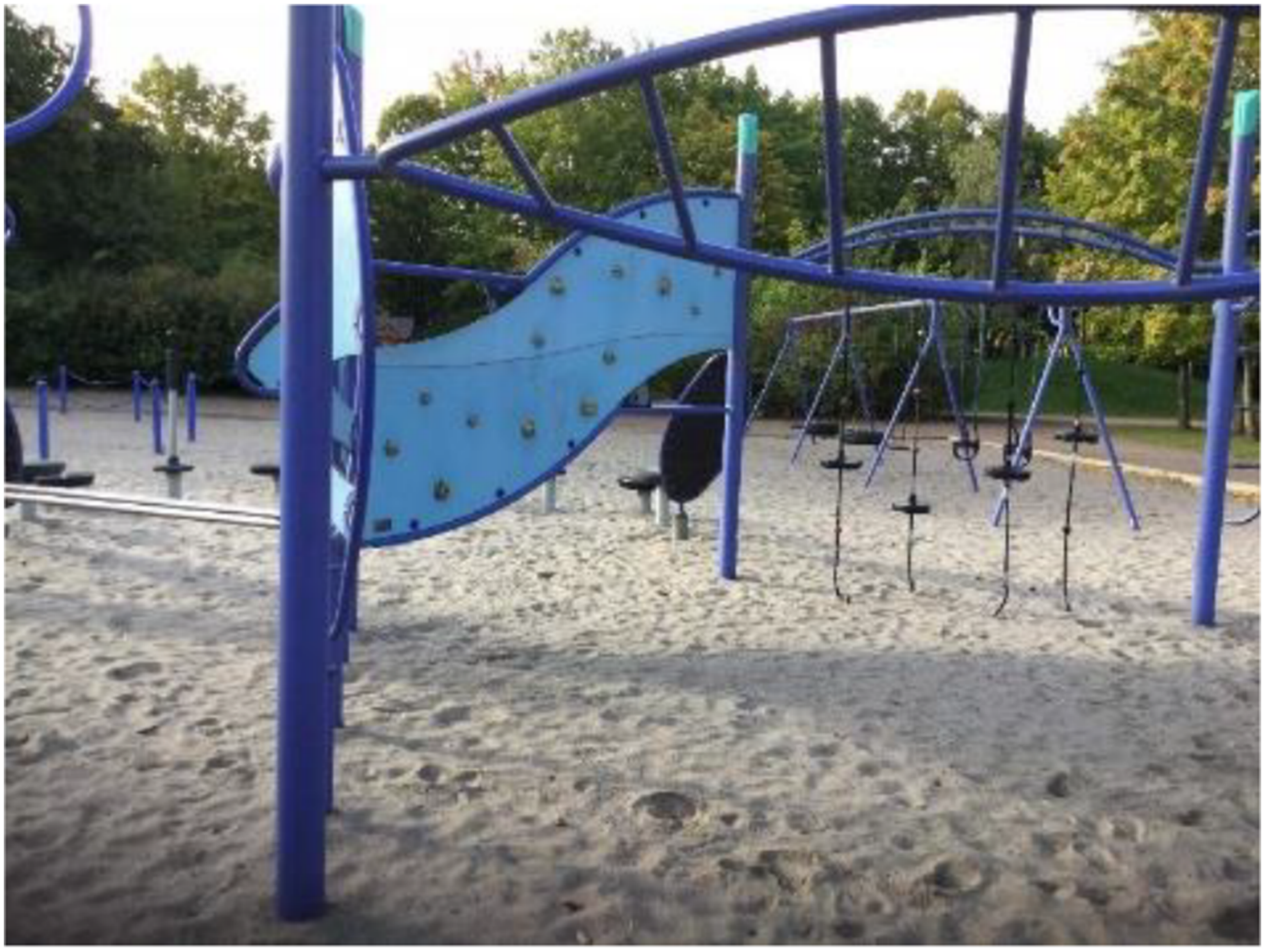
A place for joint activities [Colour figure can be viewed at wileyonlinelibrary.com]

**FIGURE 9:**
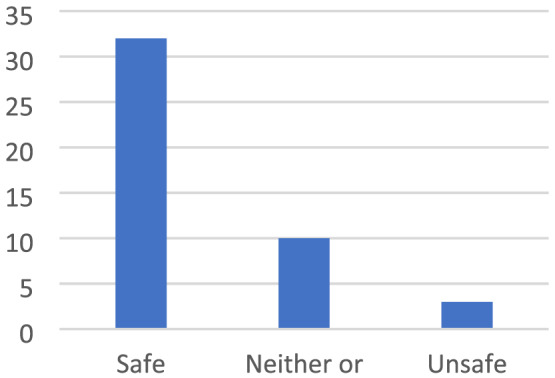
Ratings related to Figure 8 of a place for joint activities [Colour figure can be viewed at wileyonlinelibrary.com]

Being in places where the children were alone or did not know anyone created insecurity, whereas being in places where they were together with others provided a feeling of safety (Figures 8 and 9).

#### 4.3.1 Doing things together with other people creates safety

Being in places where family or friends were present felt safe. Likewise, it felt safe to be close to home or close to ‘nice’ adults. Being in school or at the playground was perceived as safe, especially if they were supervised by family members or other people they trust, ‘You can play there with your friends and swing together’. Some public places, such as the library and shops, also felt safe because there were people they could trust. The children described ‘eating together’ as a situation where they felt involved and included. Having a party or picnic were such examples: ‘There is food there, it feels safe’. To improve safety, the children suggested places for children and adults to be in together. The children also suggested having more parties and eating together, ‘Barbeque—so people will get there’, and more food retail outlets with food and sweets, ‘An ice cream shop’.

#### 4.3.2 Feeling alone creates insecurity

The children described a feeling of fear and insecurity in places where they were alone and unattended, ‘It's a lonely place’. They highlighted places in the residential area where they felt unsafe because no one could see them. Another example that was stressed was the fear of being alone in the forest. Admittedly, the children thought that the forest could be a cosy place to play and, for example, build huts in, but at the same time, they were afraid if they were there alone. The fear was related to a feeling of loneliness and the fact that there was no one else there: ‘There are few people there’. In the forest, shrubs and trees that gave shade were described as scary. In addition, the children said that they were afraid that someone would follow them and that something bad could happen to them in the forest.

### 4.4 Places where the children are not exposed to danger

**FIGURE 10:**
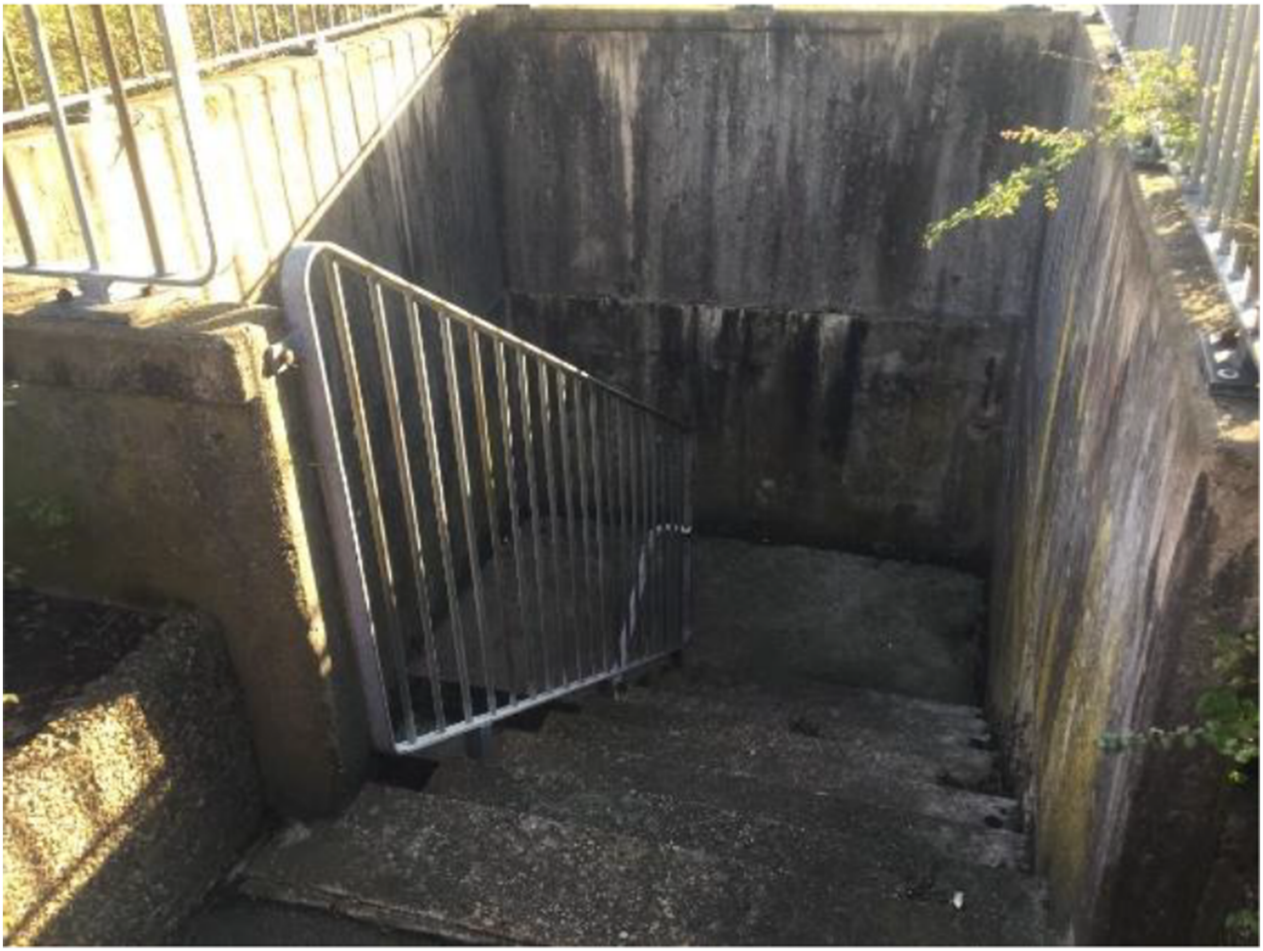
A dangerous place [Colour figure can be viewed at wileyonlinelibrary.com]

**FIGURE 11:**
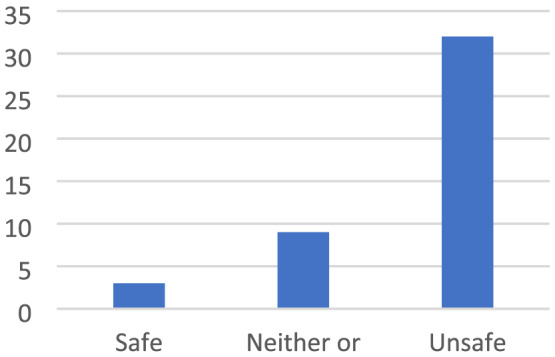
Ratings related to Figure 10 of a dangerous place [Colour figure can be viewed at wileyonlinelibrary.com]

Being in places where the children could be exposed to dangers was described as unsafe. It could be places where they might be exposed to ‘bad’ people or where they could hurt themselves (Figures 10 and 11).

#### 4.4.1 A dark forest is creepy and creates insecurity

The children described how they were scared when they were in narrow and dark places, as well as places with many bushes and trees: ‘It feels scary and deserted’. Being outside when it is dark, at night or in places where no light enters was described as scary: ‘It's scary because it's dark’. The children described a fear of places where they could be followed or places where they could be exposed to ‘bad guys’. To feel safer, the children suggested putting up lights: ‘More lights’. Further, they suggested making places lighter by removing trees and bushes: ‘You can remove trees so no one can hide’.

#### 4.4.2 It is scary to visit places you have been warned against going to

The children talked about places where they must not go, adults having warned them against those places. The reason was that there could be ‘bad’ people, shootings and gatherings of criminals: ‘Bad people there’. These places were mainly in the downtown area: ‘Not good for children to go there themselves, criminals’. The children stated that they did not know if it was dangerous, but they were afraid to go there and were told not to, and therefore they would not. To feel safe, the children suggested organizing the environment for them to be observed. They also suggested more surveillance, cameras and policemen: ‘If the police stand there you can go straight there’.

#### 4.4.3 It is unsafe to engage in activities that can hurt you

The children also felt unsafe about visiting places where they could be hurt. They talked about the risk of tumbling and hurting themselves on the gravel: ‘When you tumble it hurts, it's gravel’. Or they could hurt themselves on stones, branches and trees: ‘You can slip on the stones’. Another danger described was places where there are cars and traffic: ‘Afraid, there will be many cars’. Therefore, the children suggested creating a safe environment by removing cars, ‘Closes so cars do not drive there’, as well as stones and gravel, ‘Remove all the stones there’.

## 5 DISCUSSION

### 5.1 Discussion of the method

The children thought taking photos using photovoice was fun, and they were enthusiastic when talking about their photographs, which suggests that the children's perspective on safety was represented. However, the children's choice of photographic objects may have been influenced by the description of safety that was presented to them. Focus groups and photography are methods that have been shown to be appropriate for children (Einberg et al., 2016), reducing the power imbalance between researchers and children (Coyne, Hayes, & Gallagher, 2009; Darbyshire, Macdougall, & Schiller, 2005). The photographs taken by the children were used as a starting point for the focus‐group discussions regarding safety (Einberg et al., 2016). In this study, children could take an unlimited number of images and then select which ones were to be displayed, rated and discussed in the focus groups. This gave the children the freedom to experiment and have fun, which the children pointed out at the end of the study. This participatory approach proved to be a useful method for capturing the child's perspective.

The number of participants in each focus group is another possible limitation. However, having a few participants in each group can be an advantage, rendering it easier for children to stay focused and be heard (Coyne et al., 2009; Gibson, 2007). The photographs opened up for discussions of aspects that were not always obvious to an adult, confirming that photographs can stimulate reflection regarding a phenomenon of interest (Coad, 2007). In the current study, children were asked to describe and discuss their photographs, which reduced the risk of misinterpretation. The transferability of the results may be questioned because this study is limited to children from a vulnerable area in Sweden.

### 5.2 Discussion of the results

This study shows, by using photovoicing and focus‐group discussions, that the children described being in places that they considered bright and beautiful, where they could engage in fun activities with others without being exposed to dangers, as safe.

#### 5.2.1 A beautiful and tidy environment

In this study, the children identified clean, nice and beautiful places as safe. There is increasing evidence that suggests that the presence of urban green space can offer benefits to human health and well‐being (Islam, Johnston, & Sly, 2020) and proven important for physical activities (Topmiller, Jacquez, Vissman, Raleigh, & Miller‐Francis, 2015). Furthermore, Nordbø, Nordh, Raanaas, and Aamodt (2019) showed that built environments could enhance well‐being and that green and open spaces in the neighbourhood affected active participation and well‐being. Children identified safe and nice places as those places considered to be quiet and fun (Ahlberg, 2011). On the other hand, children in this study stated that an untidy and dirty place made them feel unsafe. Thus, an untidy school environment affected the children's sense of safety (Ahlberg, 2011). Also, youth in another study found vacant and empty spaces ‘dangerous’ and ‘unhealthy’, and places such as deserted supermarkets or playgrounds were considered unhealthy and unsafe (Petteway, 2019). Children in this study pointed out not only the importance of a clean environment but also that it is the adults' responsibility to make the environment clean and suitable. Children's right to live and grow up in a clean environment is not always respected (Humanium, 2020), and environmental issues as pollutants and noise have been claimed to have a direct effect on children's cognitive and socioemotional development (Evans, 2006; Ferguson et al., 2013). Therefore, targeted action is needed to better protect the most vulnerable populations, including children and those who are already disadvantaged in terms of socio‐economic situation or age, as they are disproportionately affected by environmental issues (Shea, 2020).

#### 5.2.2 Possibilities for having fun and playing together

Participating in meaningful activities, including organized activities, unstructured play, recreational activities or various forms of physical activities, is stated by Article 31 in the UNCRC (UN, 1989) and has several positive effects on children's development (Nordbø, Nordh, et al., 2019). The urban environment presents significant health challenges for children, such as discouraging play and physical exercise (Islam et al., 2020). In this study, the children described having meaningful activities together with other children (and adults). School‐aged children have, in another study, described leisure activities as joint and meaningful activities (Ahlberg, 2011). Participating in activities has often been related to health, well‐being and safety for children (Nordbø, Raanaas, Nordh, & Aamodt, 2019). Also, preschool‐aged children have described safety as good peer relations and meaningful activities (Basa & Kerstof, 2019). In this study, the children expressed that the playground was a safe place. The design of playgrounds has not always proved successful in meeting children's needs in terms of play and meaningful experience. Having a green outdoor space, such as a park, facilitates and predicts active participation among 8‐year‐olds in Norway (Nordbø, Nordh, et al., 2019).

Involvement in different organized activities has been associated with increased academic achievements, social relationships, life satisfaction and better mental health (Badura, Madarasova Geckova, Sigmundova, van Dijk, & Reijneveld, 2015; Breistøl, Clench‐Aas, Van Roy, & Kjærsti Raanaas, 2017; Eime, Young, Harvey, Charity, & Payne, 2013). Playgrounds and sports fields in the neighbourhood have been pointed out as the strongest predictor for active participation with friends and peers (Nordbø, Raanaas, et al., 2019). However, children living in vulnerable areas have been shown to be less engaged in organized activities (Simpkins, Delgado, Price, Quach, & Starbuck, 2013), which makes those children doubly victimized in their striving for equal health and well‐being.

#### 5.2.3 Balancing child protection with freedom of movement

In this study, the children talked about places where they must not go, adults having warned them about the risks. In this way, the adults restricted the children's freedom to move around in the local environment. The children stated that they themselves did not know if it was dangerous but respected their parents' warning. According to these children, the reason was that there could be ‘bad’ people and that there were shootings and criminals. The local environment, as part of the ecological systems (Bronfenbrenner, 1979, 2005), is important for the children's lives in terms of socialization, health and development. However, parents often restrict their children from moving freely in the local environment, something that parents see as a protective measure to safeguard their children from risk (Berggren, 2012). Parents also voice a constant need for balancing between safety, risks and other factors, such as development, independence and social acceptance (Bilker, 2014). In a study on parents' recollection of their own childhood outdoor play compared with contemporary parenting practices, a great discrepancy is shown between their recollection of idyllic special freedom and the restrictions they place on their children (Rixon, Lomax, & O'Dell, 2019). Parents' perceptions of neighbourhood safety have been positively associated with children's social–emotional development and general health (Christian et al., 2015). The UNCRC provides that children have the right to life, development (Article 6), play and leisure (Article 31). At the same time, they must be protected from violence and injuries (Article 5) (UN, 1989). When the child is part of public life and thus more exposed to the outside world, being deprived of parental protection, this can lead to greater vulnerability and put the child at risk of having their rights violated (Gerell et al., 2020). Children in this study expressed that they felt safe when they were monitored by ‘nice adults’.

A study on the boundaries of children's neighbourhoods showed that the children spent over half of their leisure time within 500 m of their homes. Children left their neighbourhood predominantly to go to other residential locations and food retail outlets (Chambers et al., 2017). Others also found that the presence of child‐relevant neighbourhood destinations and services were positively associated with early child development domains of physical health and well‐being and social competence (Christian et al., 2015). Chambers et al. (2017) also found that children spent more time at food retail outlets than at structured sport and outdoor recreation locations, which corresponds well with the statements of children in this study, who pointed out places with adults, such as the food retail outlets, as safe places.

Children's freedom of movement and their opportunities to discover their surrounding environment on their own have been reduced over the last decades. There is a trend for children to engage increasingly in sedentary activities in their spare time and for more time spent indoors. This may, in the future, lead to an increase in chronic diseases, such as type 2 diabetes, obesity, osteoporosis and depression (Faskunger, 2008; Islam et al., 2020). Children's needs are not met by simply exposing them to isolated environments such as playgrounds and the schoolyard; they need opportunities to independently explore and discover their surroundings, their neighbourhood and, as they get older, their city or landscape (FHI, 2007; UN, 1989).

Playgrounds and other outdoor environments are very important places for children's free play, physical activity and development (FHI, 2007). However, many of these places are run‐down and in need of renovation (Gerell et al., 2020), something which was also expressed by children in this study. Outdoor environments designed for children's play should include natural, interactive features and be relatively large (FHI, 2007). In this study, the children described some places as ‘too scary to visit’ because of ‘dangerous people’ or poor lighting. Inadequate lighting is something that acts as a barrier to free play, physical activity and the joy of discovery (FHI, 2007), and it might thus be possible to improve safety by better lighting. Obstacles to children's freedom of movement are largely due to community planning, and safe outdoor environments that support children's activities can be created (Faskunger, 2008). It is about well‐planned playgrounds, schoolyards and parks, as well as safe roads for walking and cycling, but also about efforts regarding the built environment to make it safe and increase children's possibility for physical and mental development, leading to health and well‐being.

## 6 CONCLUSION

In this study, safe environments in the eyes of 9‐year‐old children have been described as places that they think are bright and beautiful, where they can do fun things with others and do not risk being exposed to dangers. To enhance the joy of discovery, physical activity and friendship and to increase safety, the children in this study suggested cleaning up the environment, making it beautiful with grass designs and flowers and painting walls and houses in nice colours. Furthermore, they suggested building features that increase the opportunities for play and activities they could engage in together with others, as well as increased protection and surveillance by, for instance, making sure there is more light through removing trees and bushes and increasing the lighting. All children have the right to protection and safety. Therefore, making areas safe for children needs to take place in all settings, in the family, child and school health services, preschool and school, and everyone who works in sports and other leisure activities for children has an extremely important role in creating safe environments for all children.

## CONFLICT OF INTEREST

There is no conflict of interest.

## AUTHOR CONTRIBUTIONS

All authors, including K. E., G. I. and M. R., participated in the design of the study. M. R., together with the health promoter, the teacher and representatives from Save the Children, conducted the data collection. K. E. carried out the content analysis together with G. I. and M. R. K. E. wrote the first version of the manuscript in collaboration with G. I. and M. R., and all authors revised, read and approved the final version of the manuscript.

## FUNDING INFORMATION

The current study was carried out as a part of ‘The health promotion platform in collaboration’ at Malmö University. The platform was funded by VINNOVA (Reg. No. 2016–00421 and 2017–01272), primarily for the establishment of the health‐promoting platform (and not for research conducted within this platform).
